# Supplementary material for: Ferroptosis-Related Gene SLC1A5 Is a Novel Prognostic Biomarker and Correlates with Immune Microenvironment in HBV-Related HCC
Source: J Clin Med. 2023 Feb 21;12(5):1715. doi: 10.3390/jcm12051715 (PMC10003624; doi:10.3390/jcm12051715)
Supplement: Supplementary file 1 [file jcm-12-01715-s001.zip › Supplemental Table S1.pdf]

Signal Pathway	*P* Value	Pearson Correlation Coefficient
EMT markers	8.18E-23	0.48
Inflammatory response	8.66E-23	0.48
Degradation of ECM	6.28E-22	0.47
Ferroptosis	6.77E-22	0.47
G2M checkpoint	1.36E-21	0.47
PI3K/AKT/mTOR pathway	5.98E-22	0.47
P53 pathway	5.02E-21	0.46
Cellular response to hypoxia	4.11E-20	0.45
Tumor proliferaton signature	3.57E-19	0.44
Ether lipid metabolism	2.37E-18	0.43
IL-10 Anti-inflammatory Sigaling Pathway	1.78E-18	0.43
ECM-relatted genes	3.17E-17	0.42
Apoptosis	9.54E-17	0.41
Mucin type O glycan biosynthesis	9.06E-17	0.41
Phosphonote and phosphinate metabolism	2.12E-15	0.40
Glycerophospholipid metabolism	4.42E-14	0.38
Glycosphingolipid biosynthesis ganglio series	4.06E-14	0.38
TGF-β	3.47E-14	0.38
Angiogenesis	1.06E-13	0.37
Inositol phosphate metabolism	1.43E-12	0.36
MYC targets	7.18E-13	0.36
Sphingolipid metabolism	7.03E-12	0.35
Genes up-regulated by ROS	3.7E-10	0.32

Neomycin kanamycin and gentamicin biosynthesis	1.03E-09	0.31
DNA replication	2.55E-08	0.28
Glycosaminoglycan biosynthesis heparan sulfate heparin	5.46E-08	0.28
Glycosaminoglycan biosynthesis chondroitin sulfate dermatan sulfate	0.000000462	0.26
Taurine and hypotaurine metabolism	0.000000311	0.26
Tumor inflammation signature	0.000000304	0.26
Pentose phosphate pathway	0.00000688	0.23
Fructose and mannose metabolism	0.0000247	0.22
Purine metabolism	0.0000224	0.22
Amino sugar and nucleotide sugar metabolism	0.0000595	0.21
Riboflavin metabolism	0.0000436	0.21
Arachidonic acid metabolism	0.000102	0.20
α-Linolenic acid metabolism	0.000463	0.18
DNA repair	0.002	0.16
Mannose type O glycan biosynthesis	0.001	0.16
Valine leucine and isoleucine biosynthesis	0.003	0.16
Glycosaminoglycan degradation	0.027	0.11
Fatty acid elongation	0.054	0.10
Galactose metabolism	0.162	0.07
Glycerolipid metabolism	0.411	0.04
Sulfur metabolism	0.594	0.03
Pyrimidine metabolism	0.65	0.02
Thiamine metabolism	0.776	-0.02

Pentose and glucuronate interconversions	0.557	-0.03
Phenylalanine tyrosine and tryptophan biosynthesis	0.531	-0.03
Ascorbate and aldarate metabolism	0.421	-0.04
Pantothenate and CoA biosynthesis	0.448	-0.04
N Glycan biosynthesis	0.356	-0.05
Porphyrin and chlorophyII metabolism	0.216	-0.06
Oxidative phosphorylation	0.149	-0.07
Folate biosynthesis	0.133	-0.08
Glutathione metabolism	0.051	-0.10
Phenylalanine metabolism	0.034	-0.11
Nicotinate and nicotinamide metabolism	0.017	-0.12
Starch and sucrose metabolism	0.005	-0.15
Linoleic acid metabolism	0.002	-0.16
Terpenoid backbone biosynthesis	0.001	-0.16
Other glycan degradation	0.001	-0.17
Other types of O glycan biosynthesis	0.001	-0.17
Glycolysis Gluconeogenesis	0.0000463	-0.21
Nitrogen metabolism	0.000022	-0.22
Pyruvate metabolism	0.000021	-0.22
Steroid biosynthesis	0.0000262	-0.22
D Glutamine and D glutamate metabolism	0.0000119	-0.23
Arginine and proline metabolism	0.00000317	-0.24
Vitamin B6 metabolism	0.00000188	-0.24
Alanine aspartate and glutamate metabolism	0.000000802	-0.25

Biosynthesis of unsaturated fatty acids	0.000000<br>353	-0.26
Drug metabolism other enzymes	0.000000<br>336	-0.26
Cysteine and methionine metabolism	0.000000<br>174	-0.27
Synthesis and degradation of ketone bodies	0.000000<br>163	-0.27
Tyrosine metabolism	1.09E-08	-0.29
Glyoxylate and dicarboxylate metabolism	2.36E-09	-0.30
Metabolism of xenobiotics by cytochrome P450	5.02E-09	-0.30
Steroid hormone biosynthesis	6.7E-11	-0.33
Retinol metabolism	2.24E-11	-0.34
Tryptophan metabolism	6.74E-12	-0.35
Drug metabolism cytochrome P450	4.62E-13	-0.36
Ubiquinone and other terpenoid quinone biosynthesis	5.92E-13	-0.36
Lipoic acid metabolism	1.55E-13	-0.37
One carbon pool by folate	1.16E-13	-0.37
Fatty acid biosynthesis	2E-14	-0.38
Citrate cycle	7.01E-16	-0.40
Histidine metabolism	1.97E-15	-0.40
Butanoate metabolism	3.12E-16	-0.41
Selenocompound metabolism	2.45E-19	-0.44
Caffeine metabolism	9.1E-21	-0.46
Propanoate metabolism	4.06E-24	-0.49
Valine leucine and isoleucine degradation	3.87E-24	-0.49
Glycosylphosphatidylinositol GPI anchor biosynthesis	1.19E-24	-0.50
Arginine biosynthesis	1.53E-25	-0.51

Glycine serine and threonine metabolism	3.44E-26	-0.51
β-Alanine metabolism	8.64E-28	-0.53
Lysine degradation	4.24E-33	-0.57
Primary bile acid biosynthesis	4.24E-37	-0.60
Biotin metabolism	3.99E-39	-0.61
D Arginine and D ornithine metabolism	2.44E-41	-0.62
Fatty acid degradation	1.7E-46	-0.65
